# Bactericidal and sterilizing activity of novel regimens combining bedaquiline or TBAJ-587 with GSK2556286 and TBA-7371 in a mouse model of tuberculosis

**DOI:** 10.1128/aac.01562-23

**Published:** 2024-02-20

**Authors:** Si-Yang Li, Sandeep Tyagi, Heena Soni, Fabrice Betoudji, Paul J. Converse, Khisimuzi Mdluli, Anna M. Upton, Nader Fotouhi, David Barros-Aguirre, Lluís Ballell, Elena Jimenez-Navarro, Eric L. Nuermberger

**Affiliations:** 1 Center for Tuberculosis Research, Division of Infectious Diseases, Johns Hopkins University School of Medicine, Baltimore, Maryland, USA; 2 TB Alliance: Global Alliance for Tuberculosis Drug Development, New York, New York, USA; 3 Global Health Medicines R&D, GlaxoSmithKline R&D Limited, Tres Cantos, Madrid, Spain; St George's, University of London, London, United Kingdom

**Keywords:** GSK2556286, *Mycobacterium tuberculosis*, mouse model, tuberculosis, bedaquiline, pretomanid, TBAJ-587, TBA-7371, TBI-223, linezolid

## Abstract

The combination of bedaquiline, pretomanid, and linezolid (BPaL) has become a preferred regimen for treating multidrug- and extensively drug-resistant tuberculosis (TB). However, treatment-limiting toxicities of linezolid and reports of emerging bedaquiline and pretomanid resistance necessitate efforts to develop new short-course oral regimens. We recently found that the addition of GSK2556286 increases the bactericidal and sterilizing activity of BPa-containing regimens in a well-established BALB/c mouse model of tuberculosis. Here, we used this model to evaluate the potential of new regimens combining bedaquiline or the more potent diarylquinoline TBAJ-587 with GSK2556286 and the DprE1 inhibitor TBA-7371, all of which are currently in early-phase clinical trials. We found the combination of bedaquiline, GSK2556286, and TBA-7371 to be more active than the first-line regimen and nearly as effective as BPaL in terms of bactericidal and sterilizing activity. In addition, we found that GSK2556286 and TBA-7371 were as effective as pretomanid and the novel oxazolidinone TBI-223 when either drug pair was combined with TBAJ-587 and that the addition of GSK2556286 increased the bactericidal activity of the TBAJ-587, pretomanid, and TBI-223 combination. We conclude that GSK2556286 and TBA-7371 have the potential to replace pretomanid, an oxazolidinone, or both components, in combination with bedaquiline or TBAJ-587.

## INTRODUCTION

During the last decade, new drugs (bedaquiline [B] and delamanid) and a new regimen (bedaquiline, pretomanid, and linezolid [BPaL]) have been evaluated in clinical trials (1–7) and approved by stringent regulatory authorities for use in the treatment of multidrug-resistant tuberculosis (TB). The BPaL regimen is the first clinically proven 6-month oral dosing regimen for the treatment of multidrug-resistant TB. However, its utility for this indication, and especially its potential utility for drug-susceptible TB, is limited by the dose- and duration-dependent toxicity of linezolid (1, 2). Furthermore, reports of resistance to bedaquiline and/or pretomanid and delamanid among the clinical isolates of *Mycobacterium tuberculosis* are emerging (8, 9). The development of novel short-course regimens with greater safety and/or greater efficacy against drug-resistant strains could increase therapeutic options, promote treatment completion, improve treatment outcomes, and prevent the emergence of further drug resistance. A recently described drug, GSK2556286 (G286 or G), acting with a novel mechanism of action, increases the activity of both BPa and BPaL in mouse models of TB (10). It is currently being evaluated in a Phase 1 trial (NCT04472897). TBA-7371 (A7371 or A) is a DprE1 inhibitor in a Phase 2 a, dose escalation, trial to evaluate the safety, early bactericidal activity (EBA), and pharmacokinetics (NCT04176250). A7371 previously showed bactericidal activity and additive activity with bedaquiline *in vitro* and in a chronic BALB/c mouse infection model of TB (11). As a monotherapy in that study, A7371 showed dose-dependent bactericidal activity, causing a 1 log_10_ CFU reduction after treatment for 1 month at a dose of 300 mg/kg/d. The addition of A7371 at a lower dose of 100 mg/kg increased the bactericidal activity of bedaquiline by approximately 1 log_10_ after 4 weeks of treatment (11). Robertson et al. (12) found that A7371 prevented bacterial growth at a dose of 50 mg/kg twice daily and had dose-dependent bactericidal activity at doses of 100 and 200 mg/kg twice daily after 2 months of treatment in a chronic C3HeB/FeJ mouse infection model. A new diarylquinoline, TBAJ-587 (S587 or S) that is more potent and has a lower cardiovascular safety risk than bedaquiline in preclinical studies (13, 14), is being evaluated in a phase 1 trial (NCT04493671). It has also been shown to retain greater activity in mice than bedaquiline against a bedaquiline-resistant *M. tuberculosis* strain with a mutation in *Rv0678* (14). Here, we evaluated the bactericidal and sterilizing activity of novel combinations of a diarylquinoline (B or S587) with G286 and A7371 in a well-established BALB/c mouse model of TB.

## RESULTS

Experiment 1 explored the contribution of G286 and/or A7371 to combinations containing B or Pa by comparing the bactericidal activity of each drug administered as monotherapy and in all two- and three-drug combinations containing either B or Pa, but not both. The mean lung log_10_ colony-forming unit (CFU) counts after 4 and 8 weeks of treatment are given in Table 1. As previously observed (15), B and B-containing regimens had greater bactericidal activity than Pa and Pa-containing regimens. The activity of A7371 monotherapy at a dose of 400 mg/kg was comparable to that of Pa. Based on previously reported results of dose-ranging monotherapy studies and those of a combination study in which an additive effect was observed when G286 was combined with the BPa combination (10), we tested G286 at a dose of 50 mg/kg. As a monotherapy, this dose did not prevent mortality in a lethal subacute infection model, as mice in this group met humane endpoints for euthanasia between 3 and 4 weeks postinfection. After 4 weeks of treatment, no significant difference was observed between any two- or three-drug regimen containing B and B alone. Likewise, no two- or three-drug regimen containing Pa was significantly different from Pa alone. However, after 8 weeks of treatment, the three-drug regimen of BGA was more active thanB (*P =* 0.0197) and BA (*P =* 0.1472), suggesting that A, and perhaps G, contributes to the activity of the regimen, although the latter difference did not reach statistical significance. Notably, the four mice receiving BG for 8 weeks had lung log_10_ CFU counts of 1.93, 1.98, 2.90, and 3.45. Due to suspicion that the emergence of resistance to bedaquiline was responsible for the divergent CFU counts observed within this arm, the four isolates from this arm underwent bedaquiline susceptibility testing via a broth microdilution assay in Middlebrook 7H9 media. Only the isolate from the mouse with the highest CFU count had a bedaquiline minimum inhibitory concentration (MIC) >0.125 µg/mL. The MIC was 0.25 µg/mL or four–eight times higher than the wild-type MIC in 7H9 media of 0.03–0.06 µg/mL. PCR amplification and sequencing of the *Rv0678* and *pepQ* genes revealed an *Rv0678* mutation (nt202 A→T, resulting in a Ser→Cys amino acid change at position 68) in this isolate, while no mutation was found in the isolate with the next highest CFU count. Isolates from the BA arm were not assessed for bedaquiline resistance because there were no outlier CFU counts. However, this does not exclude the possibility of selective amplification of resistant mutants occurring in this arm as well.

**TABLE 1:** *M. tuberculosis* lung burden in mice after treatment with single or multiple drugs in Experiment 1^*b*^^,*c*,*d*^

	Mean lung log_10_ CFU counts ± SD		
Regimen	D0	W4	W8
Untreated	8.16 ± 0.16		
G_50_		^*a*^	NT
A_400_		6.74 ± 0.07	NT
Pa_100_		6.76 ± 0.15	NT
PaG		6.46 ± 0.17	5.31 ± 0.21
PaA		6.81 ± 0.26	5.35 ± 0.15
PaGA		6.65 ± 0.14	5.54 ± 0.07
B_25_		4.60 ± 0.08	NT
BG		4.33 ± 0.13	2.57 ± 0.74
BA		4.38 ± 0.24	2.14 ± 0.08
BGA		4.49 ± 0.41	1.48 ± 0.38

^*a*^ Mice met humane endpoint criteria prior to W4 due to disease progression.

^*b*^ G (GSK2556286), Pa (pretomanid), A (TBA-7371), and B (bedaquiline).

^*c*^ Subscripts indicate the dose in mg/kg.

^*d*^ N=4 mice per arm per time point; NT, not tested.

To better understand the A7371 dose–response relationships and pharmacokinetic (PK) and pharmacodynamic (PK/PD) parameters most closely associated with activity, the impact of dose and dose scheduling on the activity of A alone and in combination with BG was assessed in Experiment 2 by using the same subacute infection model used in Experiment 1. A satellite multidose PK study mimicking the doses and dose schedules of A7371 was performed on uninfected BALB/c mice and was used to estimate the A7371 PK/PD parameters in infected mice (Table 2). A7371 plasma exposures increased proportionally with dose over most of the dose ranges tested. Above 100 mg/kg, exposures increased somewhat less than proportionally, likely due to reduced oral bioavailability. A7371 monotherapy displayed dose-dependent efficacy with once daily (qd) dosing. Mice receiving 50 and 100 mg/kg qd met humane endpoints during the fourth week of treatment, whereas the doses of 200 and 400 mg/kg qd had bactericidal effects.

**TABLE 2:** Estimated PK and PK/PD parameters of TBA-7371 in Experiment 2

TBA-7371 dosing arm	Weekly dose (mg/kg)	Cmax (ng/mL)	AUC (ng.h/mL)	Weekly AUC (ng.h/mL)	T_>MIC_ (fraction of 168 h)
25 mpk bid	250	17733	37414	187070	0.177
50 mpk bid	500	25633	74914	374570	0.317
100 mpk bid	1,000	35533	144926	724630	0.391
50 mpk qd	250	29400	42749	213745	0.193
100 mpk qd	500	35367	73296	366480	0.261
200 mpk qd	1,000	55533	127534	637670	0.340
400 mpk qd	2,000	50300	242458	1212290	0.490

**Fig 1:**
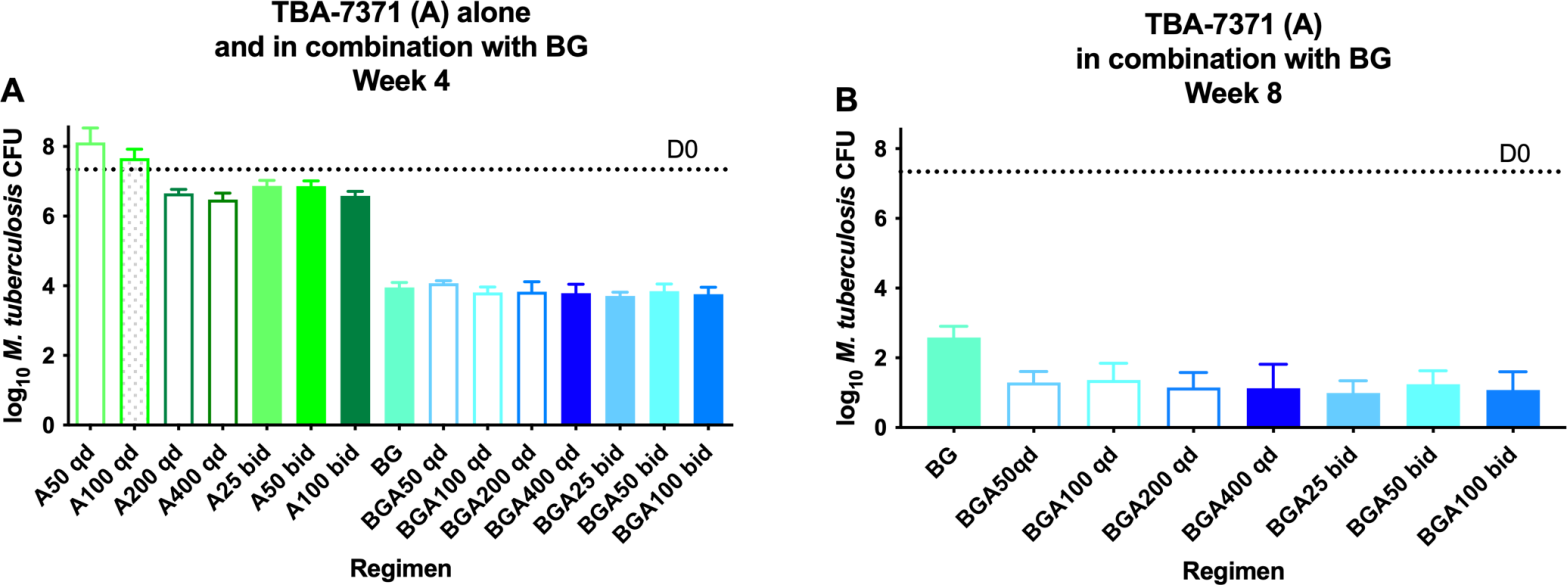
Efficacy of TBA-7371 (A) at different doses and in combination with bedaquiline (B) and GSK2556286 (G) against *M. tuberculosis* in mouse lungs after 4 (**A**) and 8 (**B**) weeks of treatment in Experiment 2. D0, Dotted line; qd, once daily; bid, twice daily.

Twice daily (bid) dosing of 25 and 50 mg/kg resulted in superior efficacy to once-daily dosing at the same total dose (50 and 100 mg/kg qd, respectively. Likewise, 100 mg/kg twice daily dosing had bactericidal activity comparable to that of 200 and 400 mg/kg once daily dosing (Fig. 1A; Table S1). These results are most consistent with the activity of A7371 monotherapy being better correlated with time above MIC (T_>MIC_) than with the maximum concentration (C_max_) or the area under the concentration–time curve (AUC). Indeed, when the data of monotherapy PK/PD were combined with similar data from an additional unpublished experiment (Fig. S1; Table S2) to determine the PK/PD parameter most closely linked to A7371 activity, the highest correlation was observed with T_>MIC_ (*R*^2^ = 0.67) compared with AUC (*R*^2^ = 0.59) and C_max_ (*R*^2^ = 0.44) (Fig. S2 to S4).

As observed in Experiment 1, the addition of A7371 to BG did not significantly increase the bactericidal activity after 4 weeks of treatment, but it did increase the bactericidal activity after 8 weeks of treatment (Fig. 1B). Remarkably, despite the dose–response relationship observed with monotherapy, all A7371 doses and dosing schedules significantly (*P* ≤ 0.0011) increased the activity of BG to a similar degree, with all BGA arms achieving a bacterial kill similar to that observed with the same regimen using a A7371 dose of 400 mg/kg once daily in Experiment 1. In order to screen for the selection of bedaquiline-resistant mutants, an aliquot representing 20% of each week 8 lung homogenate was plated directly onto an agar plate containing bedaquiline at 0.06 µg/mL. No homogenate from mice receiving a BGA combination showed CFU on bedaquiline-containing plates. The same was true for mice receiving BG, except that one of five mice in this arm had a single colony isolated on the bedaquiline-containing plate, representing approximately 0.67% of the total CFU count recovered on drug-free plates. The bedaquiline MIC against this isolate was 0.25 µg/mL, four–eight times higher than that of the wild-type MIC.

Following the demonstration of the additive bactericidal activity of the BGA regimen, Experiment 3 evaluated its sterilizing activity, with and without the addition of linezolid, with a comparison to the first-line RHZ (rifampicin + isoniazid + pyrazinamide) regimen and the BPaL regimen as controls. Based on the results of Experiment 2, the A7371 dose was lowered to 50 mg/kg once daily. A study arm receiving the 2-drug BA combination was added to confirm the contribution of G to the bactericidal activity of the 3-drug regimen. The study arm receiving the 2-drug BL combination was added to compare the respective contributions of Pa and GA when added to this 2-drug combination. Data for the untreated, RHZ, BGL, and BPaL control arms were published previously (10). After 4 weeks of treatment, the BGA regimen was significantly (*P <* 0.0001) more active than the RHZ regimen but not BPaL; BGL was not as active as BGA at Week 4 (*P =* 0.001), but there was no significant difference at Week 8. BGA plus L (BGAL) was not more active than BGL at either time point (Table 3). After 8 weeks of treatment, the BGA regimen was still more active (*P =* 0.0056) than the RHZ regimen but was significantly less active than the BPaL regimen (*P <* 0.0001). The BGA regimen resulted in significantly (*P =* 0.04) lower CFU counts compared to the BA regimen after 4 weeks of treatment. The mean CFU count after 8 weeks of treatment was also lower with the addition of G286, but the difference was not statistically significant due to the high variability in the BA arm at this time point.

**TABLE 3:** Efficacy of novel regimens combining GSK2556286 and TBA-7371 with bedaquiline, with or without linezolid, in Experiment 3^*d*^^,^^*e*^^,^^*f*^

	Mean lung log_10_ CFU counts ± SD				Proportion of mice relapsing		
Regimen	W-2	D0	W4	W8	W8 (+12)	W12 (+12)	W16 (+12)
Untreated	3.94 ± 0.15	7.30 ± 0.10		–	–	–	
R_10_H_10_Z_150_			5.12 ± 0.14	2.55 ± 0.07	–	–	7/15 (47%)
B_25_Pa_100_L_100_			3.10 ± 0.17	0.00 ± 0.00	4/15 (27%)	1/15 (7%)	
BL			4.28 ± 0.10	2.75 ± 0.52			
BGL			4.16 ± 0.34	1.91 ± 0.33	14/15 (93%)	2/14^*a*^ (14%)	1/14^*a*^ (7%)
BA_50_			3.65 ± 0.12	2.41 ± 0.93			
BG_50_A			3.24 ± 0.28	1.78 ± 0.04	7/13^*b*^ (54%)	1/15 (7%)	1/15 (7%)
BGAL			3.40 ± 0.25	1.80 ± 0.15	8/14^*c*^ (57%)	1/13^*c*^ (8%)	0/15 (0%)

^*a*^ One mouse died due to a gavage accident and could not be assessed for relapse.

^*b*^ Two mice lost due to non-experimental causes.

^*c*^ Total of three mice lost due to non-experimental causes.

^*d*^ R (rifampin), H (isoniazid), Z (pyrazinamide), G (GSK2556286), Pa (pretomanid), A (TBA-7371), and B (bedaquiline).

^*e*^ W4, W8 +week 4 and week 8. W8 + (12), W12 + (12), W16 + (12) =treatment for indicated number of weeks followed by 12 weeks with no treatment.

^*f*^ *N* = 6 mice per arm at W-2 and D0; *N* = 4 mice per arm at W4 and W8, *N* = 15 mice per arm for relapse time points Subscripts indicate the dose in mg/kg.

Regarding sterilizing activity, after 8 weeks of treatment, a lower proportion of mice relapsed in the BPaL arm (27%) compared to the BGA arm (54%) and BGAL arm (57%), but the difference was not statistically significant. BGAL treatment did result in a significantly (*P =* 0.0352) lower proportion of relapses than BGL treatment at this time point. After 12 weeks of treatment, one or two mice relapsed in all four arms. After 16 weeks of treatment, one mouse relapsed in the BGA and BGL groups, whereas 7/15 mice relapsed in the group treated with RHZ (*P =* 0.0352). The significantly lower proportion of relapses after 12 and 16 weeks of treatment with BGA compared to 16 weeks of treatment with RHZ demonstrates the superior sterilizing activity of BGA compared to that of the first-line regimen. Treatment with the four-drug combination (BGAL) resulted in no relapses after 16 weeks of treatment (0/15), but this is not significantly different from the proportion of relapses observed with BGA treatment.

Therefore, the addition of linezolid did not increase the bactericidal or sterilizing activity of the BGA regimen, and the bactericidal activity of the 4-drug regimen was greater than that of BL alone, confirming the contribution of GA to the 4-drug regimen.

**Fig 2:**
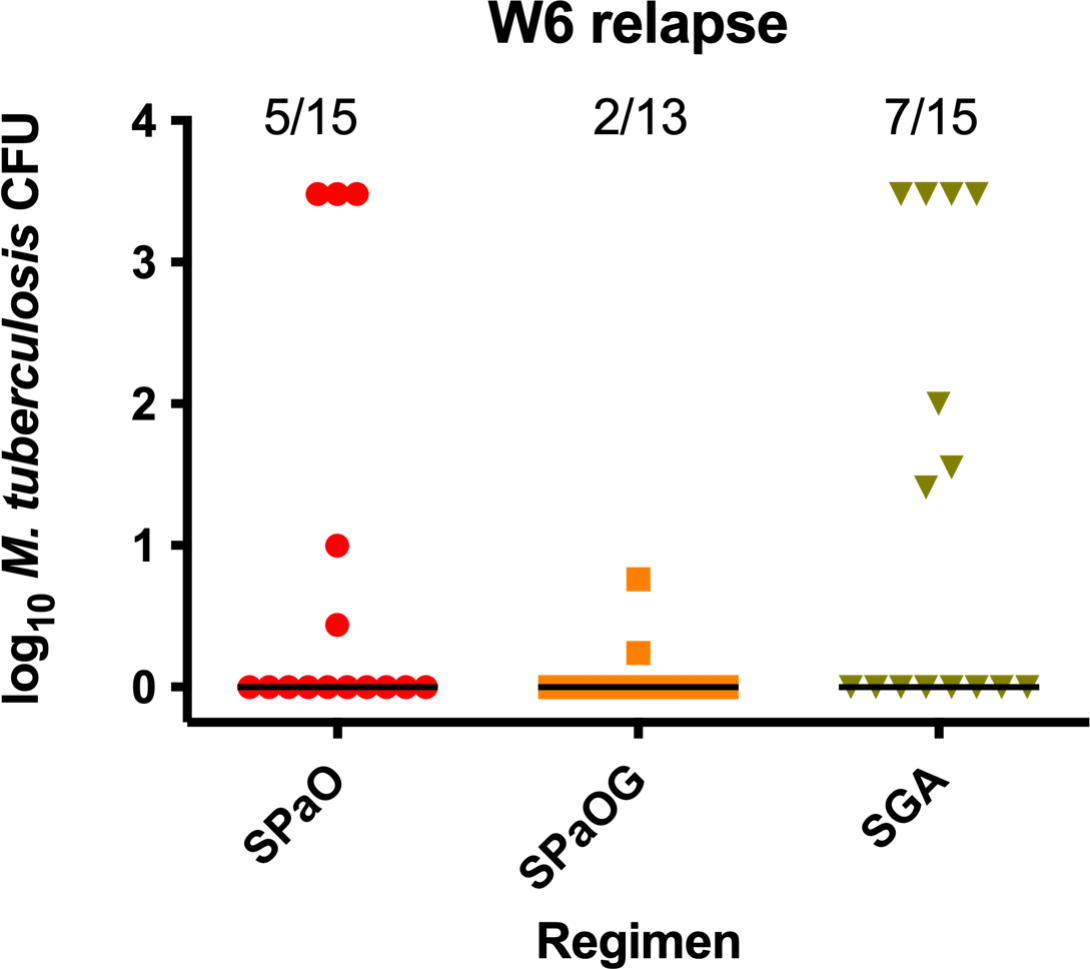
Bacterial burden and proportion of mice relapsing after treatment for 6 weeks with S587 + pretomanid + TBI-223 (SPaO), SPaO + GSK2556286 (SPaOG), or S + TBA-7371 + G (SGA) followed by 12 weeks of no treatment. For graphical representation, mouse lungs with too many bacteria to count were arbitrarily assigned a value of 3.48 log_10_.

Experiment 4 evaluated the sterilizing activity of GA when combined with the next-generation diarylquinoline TBAJ-587 (S587) instead of bedaquiline. This regimen was compared to a novel BPaL-like regimen in which S587 replaced bedaquiline and a next-generation oxazolidinone, now in phase 2 trial (TBI-223 or O) replaced linezolid. The latter regimen has sterilizing activity as good or better than that of BPaL in this mouse model (16). Because a prior study in this model showed that the addition of G286 to BPaL lowered the lung CFU counts and the proportion of mice relapsing (albeit in a non-statistically significant fashion) (10), a study arm evaluating the effect of adding G286 to S587PaO was also added. As expected, untreated mice reached humane endpoints and required euthanasia 21 days after infection or 7 days after treatment initiation. All combination regimens exhibited strong bactericidal and sterilizing activity. After 4 weeks of treatment, the addition of G286 to the S587PaO regimen significantly (*P =* 0.0324) increased the bactericidal activity and the combination of S587GA showed comparable activity to that seen with S587PaO (Table 4). Relapse assessments showed that, compared to S587PaO, S587PaOG treatment resulted in lower proportions of mice relapsing at both time points, although the differences were not statistically significant. It is also noteworthy that the bacterial burden in three of the five mice relapsing in the S587PaO arm after 6 weeks of treatment was orders of magnitude higher than that in the two mice relapsing in the S587PaOG arm (Fig. 2), further suggesting the contribution of G286 to the regimen. There were no statistically significant differences in the proportions of mice relapsing in the arms receiving S587PaO and S587GA, although the proportions of relapse were modestly lower in the S587PaO arm.

**TABLE 4:** Impact of addition of GSK2556286 to the bactericidal and sterilizing activity of a novel TBAJ-587, pretomanid, and TBI-223 regimen and comparison to another regimen of TBAJ-587, TBA-7371, and GSK2556286 in Experiment 4^*d*^

	Mean lung log_10_ CFU counts ±SD			Proportion of mice relapsing	
Regimen	D-13	D0	W4	W6(+12)	W9(+12)
Untreated	4.25 ± 0.05	7.58 ± 0.14	9.19 ± 0.28^*a*^		
S587_25_Pa_100_O_100_			2.10 ± 0.14	5/15	1/15
S587PaOG_50_			1.48 ± 0.47	2/13^*b*^	0/15
S587GA_400_			2.12 ± 0.22	7/15	2/14^*c*^

^*a*^ All mice from the untreated group reached humane endpoints 21 days after infection. Three of five untreated mouse lungs were plated for CFU determinations.

^*b*^ Two mice died during the follow-up period after treatment and could not be assessed: One mouse reached humane endpoints 11 weeks after completing treatment, while the other had no preceding signs of illness.

^*c*^ One mouse died without preceding signs of illness 8 weeks after treatment completion and could not be assessed. D-13 = CFU implanted in the lung after aerosol infection.

^*d*^ D0 = CFU at treatment initiation. W4, =week 4. W6 + (12), W9 + (12) =treatment for the indicated number of weeks followed by 12 weeks of no treatment. TBAJ-587 (S587), pretomanid (Pa), TBI-223 (O), TBA-7371 (A), and GSK2556286 (G); *N* = 6 mice per arm at D-13 and D0; *N* = 5 mice per arm at W4, *N* = 15 mice per arm for relapse time points. Subscripts indicate the dose in mg/kg.

## DISCUSSION

The experiments presented here demonstrate that a novel 3-drug combination of bedaquiline, G286, and A7371 has a strong bactericidal and sterilizing activity that is superior to that of the first-line RHZ regimen and comparable to that of the BPaL regimen in a well-established mouse model of TB. The BPaL regimen has proven clinically successful in treating extensively drug-resistant TB and treatment-refractory multidrug-resistant TB following its initial discovery using this model (15). However, its utility has been limited by the toxicity of the linezolid component (1, 2, 17, 18), which precludes any consideration for widespread use to treat drug-susceptible TB. In addition, both baseline and acquired resistance to pretomanid and delamanid have been reported in clinical isolates, albeit rarely (1, 8, 19). The promising efficacy of the BGA combination identified here suggests that the replacement of both pretomanid and linezolid in the BPaL regimen by drugs with completely different modes of action may retain its superiority to the first-line regimen. This combination may warrant clinical evaluation as a novel backbone for a pan-TB regimen if G286 successfully completes its ongoing evaluation in phase 1 trials (e.g., NCT04472897). The results of a phase 2 a trial (NCT04176250) evaluating the EBA of TBA-7371 were recently reported. Dose-dependent EBA was observed, with a maximum EBA of −0.13 log_10_CFU/mL/day in the arm receiving 100 mg thrice daily, reflecting an observed association of greater bactericidal activity with divided daily dosing and higher C_min_ and AUC, as we observed in mice. With respect to dose translation, we note that the plasma AUC_0-24h_ values of 43 and 242 µg.h/mL observed in mice receiving A7371 doses of 50 and 400 mg/kg/day, respectively, are similar to the AUC_0-24h_ values of 44 and 175 µg.h/mL observed after receiving doses of 100 and 400 mg, respectively, in thephase 1 single ascending dose trial (20), and AUC_0-24h_ values of 38 and 142 µg.h/mL were observed after 14 daily doses of 100 and 400 mg, respectively, in the EBA trial. Therefore, the A7371 doses used in mice in this study are reasonable representations of the dose range evaluated in the EBA trial (21).

Baseline and acquired resistance to bedaquiline in clinical isolates are being reported, driven primarily by mutations in *Rv0678* (8, 9), which are associated with relatively small shifts in bedaquiline MIC. Bedaquiline exerts strong selective pressure in the mouse model used in the current study. Indeed, prior studies have demonstrated selective amplification of bedaquiline-resistant mutants in mice despite co-administering bedaquiline with TB drugs with established clinical efficacy, such as linezolid or moxifloxacin (22). Despite its additive activity when combined with bedaquiline, G286, as a lone companion drug, was not sufficient to fully prevent selective amplification of bedaquiline-resistant mutants during co-administration as BG, as demonstrated by the emergence of bedaquiline resistance in one of four mice in Experiment 1 and in one of five mice in Experiment 2. The addition of A7371 to BG prevented the selection of bedaquiline-resistant mutants. However, this was not its sole contribution to the regimen, as A7371 made a significant contribution to the bactericidal activity of the three-drug regimen in Experiments 1 and 2 despite the fact that most mice treated with BG did not harbor bedaquiline-resistant isolates. Whether the two-drug combination of BA also would select for bedaquiline-resistant mutants was not assessed in the present study. However, we recently found that loss-of-function mutations in *Rv0678* cause small reductions in the susceptibility of *M. tuberculosis* to A7371 in relation to somewhat larger effects on susceptibility to other DprE1 inhibitors in clinical development and to bedaquiline (23). Therefore, it seems likely that BA would also selectively amplify such mutants. Therefore, it would be prudent to seek a fourth drug that further increases the activity of the BGA combination and/or further reduces the risk of amplifying resistance. The addition of linezolid did not increase the bactericidal or sterilizing activity of the three-drug regimen in Experiment 3. Additional preclinical studies are underway to evaluate other potential companion drugs.

A recent study showed that the replacement of bedaquiline in the BPaL regimen with the more potent next-generation diarylquinoline, S587, increases the bactericidal activity of the regimen against both wild-type *M. tuberculosis* and *Rv0678* mutants (14). We have also shown that a novel BPaL-like regimen in which S587 replaces bedaquiline and the next-generation oxazolidinone TBI-223 replaces linezolid has sterilizing activity as good or better than that of BPaL (16). Therefore, the finding in Experiment 4 that the combination of GA can replace PaO in combination with S587 without loss of efficacy suggests that the former drug pair may still be effective companions with more potent next-generation diarylquinolines. Additional studies with the other diarylquinoline now entering phase 2 trials, S876, are ongoing.

There are limitations in the present study. Although the sub-acute, high-dose aerosol infection BALB/c mouse model of tuberculosis is valuable in predicting the relative clinical efficacy of new antituberculosis compounds and combined regimens, the lung lesions in this strain of mice are not representative of the necrotic caseous lesions and cavities found in human pulmonary tuberculosis. Such lesions are associated with higher bacterial burdens, differential drug partitioning into caseum, and differing microenvironments that may affect bacterial phenotypic susceptibility and drug effects (24–27). C3HeB/FeJ mice develop a range of lesion types, including caseous lung lesions and even cavities (28–30), and may be valuable for validating results from BALB/c mice. To date, bedaquiline-containing regimens have shown strong bactericidal and sterilizing activity in C3HeB/FeJ mice (12, 24, 29, 30), and both G286 and A7371 have demonstrated activity as monotherapy. Further studies to verify the results observed here with novel regimens based on a backbone combining these three drugs in the potentially more informative but also more complicated and costly C3HeB/FeJ mouse model are warranted.

We experienced a few non-experimental losses of mice, which could have conceivably influenced the interpretation of our findings by reducing the number of observed events, such as relapse. However, even if all missing mice in the BGA and BGAL arms of Experiment 3 were assumed to relapse (or not relapse), it would not have changed the outcomes inferred from the statistical testing of these regimens compared to RHZ or BPaL. In contrast, if both missing mice receiving BGA or the one missing mouse receiving BGAL for 8 weeks were assumed to relapse, the *P* value for the comparison of either regimen with BGL would increase from <0.05 to 0.08, reducing the confidence with which these regimens could be declared to have different effects from each other.

Prior studies using this model have shown that the addition of G286 increases the bactericidal and sterilizing activity of bedaquiline+pretomanid (10), as does the addition of moxifloxacin (manuscript in preparation). Given this and the recent evidence from the TB-PRACTECAL trial (31) that the addition of moxifloxacin to BPaL increases the rate of sputum culture conversion, studies evaluating the incorporation of pretomanid and/or moxifloxacin into the diarylquinoline +G286 +A7371 combination are also ongoing.

## MATERIALS AND METHODS

All housing conditions and procedures involving mice were approved by the Institutional Animal Care and Use Committee at Johns Hopkins University School of Medicine and were carried out in accordance with the GSK Policy on the Care, Welfare, and Treatment of Animals.

### Mice

Female-specific pathogen-free BALB/c mice aged 5–6 weeks were purchased from Charles River (Wilmington, MA). Mice were housed in a bio-safety level 3 animal facility with access to food and water provided *ad libitum*.

### MIC testing

The broth macrodilution method using Middlebrook 7H9 broth with 10% oleic acid–albumin–dextrose–catalase (OADC) (Fisher, Pittsburgh, PA) without Tween 80 using polystyrene tubes and drug concentrations in doubling dilutions was used to determine the MICs against the *M. tuberculosis* H37Rv strain used to infect mice. The concentration range of bedaquiline used was 0.0017–1.0 µg/mL.

### PCR and DNA sequencing

Genomic DNA from *M. tuberculosis* H37Rv was extracted from bedaquiline-resistant isolates using the cetyltrimethylammonium bromide (CTAB)-NaCl method (32). To amplify and sequence the *Rv0678* gene, forward primer *Rv0678*-F (5’ CCTCTGCCGCATGAAGTT 3’) and reverse primer *Rv0678*-R (5’ TGGTCACTCCTTGTTGATGC 3’) were used.

### Mycobacterial strain

*M. tuberculosis* H37Rv was mouse-passaged, frozen in aliquots, and subcultured in Middlebrook 7H9 broth with 10% OADC and 0.05% Tween 80 prior to infection.

### Infection

BALB/c mice were infected with *M. tuberculosis* H37Rv, using the Inhalation Exposure System (Glas-col, Terre Haute, IN). The subacute infection model of BALB/c mice was initiated with a late log-phase culture in 7H9 broth (optical density at 600 nm of 0.8–1) with the goal of implanting approximately 4 log_10_ CFU in the lungs. Mice were randomized to different treatment groups and held for 2 weeks before beginning treatment.

### Drug treatments

GSK2556286 was formulated in 1% methylcellulose. TBA-7371 was prepared in 0.5% carboxymethylcellulose with 0.1% Tween 80. TBA-7371 was administered twice daily (8 h apart) in some arms of Experiment 2, as indicated. Other drugs were formulated and administered at the indicated dose as previously described (15, 33): rifampin and isoniazid (both at 10 mg/kg) and pyrazinamide (150 mg/kg) in distilled water, bedaquiline (25 mg/kg) or TBAJ-587 (25 mg/kg) in acidified 10% (2-hydroxypropyl)-ß-cyclodextrin (HPCD) solution, pretomanid (100 mg/kg) in 20% HPCD and lecithin micelle (CM-2) formulation, and linezolid (100 mg/kg) in 0.5% methylcellulose. Drugs were administered orally via gavage once daily, 5 days per week in all experiments. The diarylquinoline and Pa were administered together followed by GSK286 an hour later and then by a fourth drug 3–4 h later.

### Assessment of efficacy

Two microbiological outcomes were assessed: lung CFU counts during treatment and the proportion of mice relapsing after completion of treatment. Lungs were collected and homogenized in glass grinders at prespecified time points during and after drug treatment. The homogenates were serially diluted in PBS and plated on Middlebrook 7H11 agar plates supplemented with 10% (v/v) OADC (GIBCO) and cycloheximide [10 mg/mL], carbenicillin [50 mg/mL], polymixin B [25 mg/mL], and trimethoprim [20 mg/mL]. Homogenates from mice receiving drug combinations were plated on the same agar media but with the addition of activated charcoal powder (0.4% w/v) to mitigate the carryover effect of bedaquiline. Colonies were counted after 4 and 6 weeks of incubation at 37°C to ensure all cultivable bacteria would be detected. Relapse after differing durations of treatment with drug combinations was assessed by holding cohorts of 15 mice per group for an additional 12 weeks without treatment before euthanasia of the mice and plating the entire lung homogenate, as described above. Relapse was defined by the detection of ≥1 CFU.

### Statistical analysis

CFU counts (*x*) were log-transformed (as *x* + 1) before analysis. Group means were compared by one-way analysis of variance with Dunnett’s posttest to control for multiple comparisons. Group relapse proportions were compared using Fisher’s exact test, adjusting for multiple comparisons. GraphPad Prism version 9 (GraphPad, San Diego, CA) was used for all analyses. The use of 15 mice per group for relapse assessment provides >80% power to detect 50 percentage point differences in the proportion of mice relapsing. Smaller differences are not expected to be meaningful in terms of predicting clinically significant differences in treatment duration required to prevent relapse.

### TBA-7371 PK/PD assessment

A repeated-dose PK study was performed on uninfected female BALB/c mice in the fed state, and plasma concentrations were determined with three animals per dosing regimen following daily (qd) oral administration of TBA-7371 at dose levels of 50, 100, 200, and 400 mg/kg/day as well as twice daily dosing (bid; 0 and 8 h) of 25, 50, and 100 mg/kg for 7 consecutive days. Blood samples were collected at 0.5, 1, 2, 4, 8, 12, and 24 h after the last dose administration. Blood samples were processed into plasma. Plasma concentrations of TBA-7371 were determined by LC-MS/MS with a limit of quantitation of 1.00 ng/mL. Non-compartmental PK parameters were determined based on the mean data using WinNonlin (versions 6.4 and 7.0). An inhibitory sigmoid Emax model with a variable slope (WinNonlin PD model 108) was used to describe the relationship between PK and PK/PD parameters (AUC, T_>MIC_, and C_max_) and the change in lung CFU counts. R-squared values were used to evaluate goodness-of-fit.

## SUPPLEMENTAL MATERIAL

Supplemental material aac.01562-23-s0001.docx Dose ranging and fractionation; PK/PD.

ASM does not own the copyrights to Supplemental Material that may be linked to, or accessed through, an article. The authors have granted ASM a non-exclusive, world-wide license to publish the Supplemental Material files. Please contact the corresponding author directly for reuse.
