# Supplementary material for: Bactericidal and sterilizing activity of novel regimens combining bedaquiline or TBAJ-587 with GSK2556286 and TBA-7371 in a mouse model of tuberculosis
Source: Antimicrob Agents Chemother. 2024 Feb 20;68(4):e01562-23. doi: 10.1128/aac.01562-23 (PMC10989019; doi:10.1128/aac.01562-23)

Table S1. Dose-ranging and dose-fractionating study to determine the optimal dose of the DprE1 inhibitor TBA-7371 (A7371) in combination with bedaquiline (B) and GSK’286 (G286)

|  | **Mean Lung log_10_ CFU Counts ± SD** | | | |
| --- | --- | --- | --- | --- |
| Regimen | W-2 | D0 | W4 | W8 |
| Untreated | 4.11±0.02 | 7.65±0.58 |  |  |
| A7371_25_ bid |  |  | 6.87±0.15 |  |
| A7371_50_ qd |  |  | 7.91±0.23***** |  |
| A7371_50_ bid |  |  | 6.86±0.15 |  |
| A7371_100_ qd |  |  | 7.58±0.28***** |  |
| A7371_100_ bid |  |  | 6.55±0.13 |  |
| A7371_200_ qd |  |  | 6.66±0.11 |  |
| A7371_400_ qd |  |  | 6.47±0.19 |  |
| B_25_G286_50_ |  |  | 3.95±0.15 | 2.58±0.33 |
| BG 286+ A7371_25_ bid |  |  | 3.71±0.11 | 0.99±0.36 |
| BG286 + A7371_50_ qd |  |  | 4.08±0.07 | 1.29±0.31 |
| BG286 + A7371_50_ bid |  |  | 3.84±0.21 | 1.24±0.38 |
| BG286 + A7371_100_ qd |  |  | 3.81±0.16 | 1.37±0.48 |
| BG286 + A7371_100_ bid |  |  | 3.76±0.20 | 1.08±0.52 |
| BG286 + A7371_200_ qd |  |  | 3.83±0.28 | 1.15±0.44 |
| BG286 + A7371_400_ qd |  |  | 3.79±0.25 | 1.41±0.31 |
| Total | 2 | 4 | 79 | 40 |
| * Mice sacrificed after 3 weeks of treatment appeared sick.  N=2 and 4 mice for W-2 and D0, respectively. N= 5 mice per arm at W4 and W8.  Subscripts indicate dose in mg/kg. | | | | |

Table S2. Dose-ranging and dose-fractionated activity of TBA-7371 (A7371). Doses in mg/kg administered daily (qd), twice daily (bid), or thrice weekly (tiw).

|  | **Mean Lung log_10_ CFU Counts ± SD** | |
| --- | --- | --- |
| Dose and frequency | D0 | W3 |
| Untreated | 7.28±0.08 |  |
| A7371_15_ bid |  | 6.88±0.04 |
| A7371_30_ qd |  | 7.67±0.16 |
| A7371_50_ tiw |  | 7.31±0.06 |
| A7371_50_ bid |  | 6.35±0.14 |
| A7371_100_ qd |  | 7.39±0.28 |
| A7371_167_ tiw |  | 7.29±0.18 |
| A7371_150_ bid |  | 7.30±0.15 |
| A7371_300_ qd |  | 6.44±0.29 |
| A7371_500_ tiw |  | 6.70±0.40 |
| A7371_300_ bid |  | 5.93±0.10 |
| A7371_600_ qd |  | 6.25±0.17 |
| N= 3 mice per arm. Mice were treated 7 days per week.  Subscripts indicate dose in mg/kg. | | |

FIG S1. Supplemental Mouse PD data for TBA-7371

FIG S2. PK/PD Correlation Plot for Weekly AUC vs. Efficacy for TBA-7371

R^2^ = 0.59


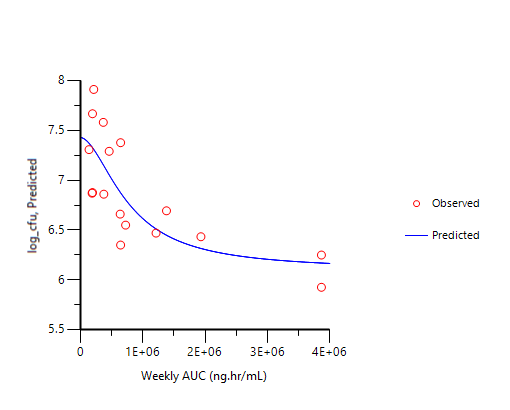


FIG S3. PK/PD Correlation Plot for Cmax vs. Efficacy for TBA-7371

R^2^ = 0.44


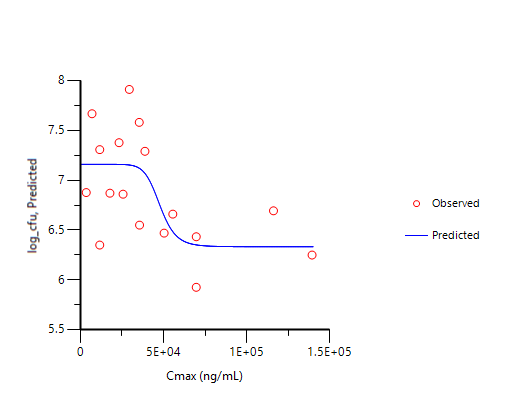


FIG S4. PK/PD Correlation Plot for Weekly T_>MIC_ vs. Efficacy for TBA-7371

R^2^ = 0.67


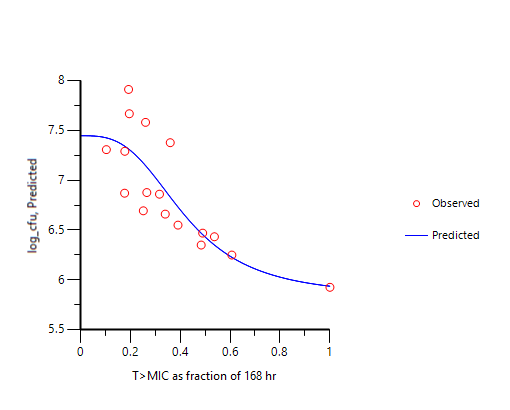

Supplement: Supplemental material — Dose ranging and fractionation; PK/PD. [file aac.01562-23-s0001.docx]
